# Altered Production and Cellular Levels of Hydrogen Sulfide (H_2_S) in Placental Trophoblasts from Pregnancies Affected by Pre-Eclampsia

**DOI:** 10.3390/pathophysiology32010010

**Published:** 2025-03-04

**Authors:** Xiaodan Chu, Jie Xu, Xinggui Shen, Wenji Sheng, Jingxia Sun, Yang Gu, David F. Lewis, Danielle Cooper, Dani Zoorob, Yuping Wang

**Affiliations:** 1 Department of Obstetrics and Gynecology, Louisiana State University Health Sciences Center, Shreveport, LA 71103, USA; 820715@hrbmu.edu.cn (X.C.); jxu001@lsuhs.edu (J.X.); 813309@hrbmu.edu.cn (W.S.); yg626@yahoo.com (Y.G.); dfl001@lsuhs.edu (D.F.L.); dsc001@lsuhs.edu (D.C.); dzo001@lsuhs.edu (D.Z.); 2 Department of Obstetrics and Gynecology, The Second Affiliated Hospital, Harbin Medical University, Harbin 150086, China; 3 Department of Pathology, Louisiana State University Health Sciences Center, Shreveport, LA 71103, USA; xsh001@lsuhs.edu; 4 Department of Obstetrics and Gynecology, The First Affiliated Hospital, Harbin Medical University, Harbin 150001, China; sjxsw2020@szu.edu.cn

**Keywords:** H_2_S, CBS, CSE, 3-MST, trophoblast, placenta, pre-eclampsia

## Abstract

Background/Objectives: Hydrogen sulfide (H_2_S) is a vasorelaxant gas and exerts anti-oxidative, anti-inflammatory, and cytoprotective effects. H_2_S has been implicated in regulating placental vaso-activity and angiogenesis. It is believed that abnormal trophoblast production of vasodilators and angiogenic factors contributes to pre-eclampsia development. However, little is known about whether aberrant H_2_S production is present in placental trophoblasts from pre-eclamptic pregnancies. Methods: Trophoblasts were isolated from normal and pre-eclamptic placentas. After incubation, cell production of H_2_S in the culture medium and the cellular levels of H_2_S were analyzed by reversed phase high-performance liquid chromatography (RP-HPLC). Expression levels of the three key H_2_S converting enzymes, cystathionine-β-synthase (CBS), cystathionine-γ-lyase (CSE), and 3-mercaptopyruvate sulfurtransferase (3-MST), were determined by immunohistochemistry. The protein expression of CBS and CSE was assessed by Western blot analysis. Results: (1) Trophoblast production and cellular levels of H_2_S were significantly reduced in cells from pre-eclamptic vs. normal placentas; (2) free H_2_S production was increased in a time-dependent manner in cultured trophoblasts from normal, but not from pre-eclamptic, placentas; and (3) strong CBS and CSE expression was seen in trophoblasts from normal, as opposed to pre-eclamptic, placentas. Reduced CBS and CSE expression in trophoblasts from pre-eclamptic vs. normal placentas were confirmed by Western blot analysis; and (4) 3-MST expression was undetachable in both normal and pre-eclamptic placentas, but 3-MST expression was strongly expressed in the first and second trimester placentas. Conclusions: These data provide plausible evidence that downregulation of CBS and CSE, but not 3-MST, expression may be responsible for reduced free H_2_S production and decreased cellular H_2_S levels in pre-eclamptic placentas. Our data provide further evidence that expression of 3-MST in placental trophoblasts is likely gestational age (developmental)-dependent.

## 1. Introduction

Pre-eclampsia is a multisystem disorder in human pregnancy, which is characterized by increased maternal blood pressure and excess urine protein excretion in women after 20 weeks of gestation. This pregnancy disorder complicates 2–8% of pregnancies and is a leading cause of maternal and fetal morbidity and mortality worldwide. Although the pathogenesis of pre-eclampsia is not fully understood, abnormal placental function remains the major contribution in the pathogenesis of pre-eclampsia, as removal of the placenta is necessary for clinical symptoms to regress. The two-stage model, i.e., incomplete spiral artery remodeling in the uterus leading to placental ischemia (stage 1), and the release of antiangiogenic factors from the ischemic placenta into maternal circulation, leading to maternal vascular endothelial injury (stage 2) [1], along with increased vasoconstriction, increased oxidative stress, and inflammatory cytokine production by placental trophoblasts [2,3,4,5], are believed to be the central pathophysiology and pathogenesis in pre-eclampsia development.

Placental trophoblasts produce numerous vaso-activators and growth factors that play critical roles in maintaining placental vascular development and functionality. It has been demonstrated that decreased vasodilator prostacyclin and nitric oxide production and increased vasoconstrictor thromboxane and endothelin production, etc., by placental trophoblasts contribute significantly to increased vasocontractility seen in placentas from women complicated with pre-eclampsia [2,6,7].

Like nitric oxide and carbon oxide, hydrogen sulfide (H_2_S) is another endogenous vasoactive gas molecule. H_2_S is produced by two pyridoxal-5′-phosphate-dependent enzymes, cystathionine-β-synthase (CBS) and cystathionine-γ-lyase (CSE) [8], from the amino acid L-cysteine, and by 3-mercaptopyruvate sulfurtransferase (3-MST), via the reaction of 3-mercaptopyruvate with dihydrolipoic acid and thioredoxin [9]. H_2_S is an important physiological mediator and exerts anti-oxidative, anti-inflammatory, and cytoprotective effects. It is believed that H_2_S may play an important role in regulating placental angiogenesis. A study by Chen et al. found that the H_2_S-producing ability of BeWo cells, a trophoblast cell line generated from the placenta from a patient with choriocarcinoma [10], was significantly inhibited by either inhibitors of CBS or CSE [11], suggesting that aberrant H_2_S production may be involved in the pathogenesis of pre-eclampsia. Downregulation of CBS and CSE expression was reported in placentas from pre-eclamptic patients [12,13]. However, cellular production and cellular levels of H_2_S have not been studied in placental trophoblasts from pre-eclampsia. The objective of the present study is to determine if placental trophoblasts produce H_2_S, and to discover which converting enzymes may contribute to aberrant placental trophoblast production of H_2_S in pre-eclampsia.

## 2. Materials and Methods

### 2.1. Chemicals and Reagents

Dulbecco’s modified Eagle’s medium (DMEM, D5523), Percoll (P1644), Hanks balanced salt solution (HBSS, H4891), Monobrombimane (MBB, B4380), and antibody against β-actin (A2066) were purchased from Sigma-Aldrich (St. Louis, MO, USA); Trypsin (LS003703) and Deoxyribonuclease (LS002139) were purchased from Worthington Biochemical Corporation (Lakewood, NJ, USA); fetal bovine serum (FBS) was from Atlantic Biologicals (Flowery Branch, GA, USA); Antibodies against CBS (12217-1-AP) and CSE (14787-1-AP) were purchased from Proteintech (Rosemont, IL, USA); and antibody against 3-MST (AB224043) was from Abcam (Waltham, MA, USA). All other chemicals were from Sigma-Aldrich, unless indicated.

### 2.2. Placenta Collection

A total of thirty-six placentas were used in this study. Third trimester/term placentas from normotensive (*n* = 18) and pre-eclamptic (*n* = 12) pregnancies were collected after delivery from the main hospital of Louisiana State University Health Sciences Center, Shreveport (LSUHSC-S), Louisiana. First trimester (*n* = 3) and second trimester (*n* = 3) placental tissues were from selective pregnancy termination at the Department of Obstetrics and Gynecology, the First Affiliated Hospital of Harbin Medical University, China. Tissue collection was approved by both institutions: LSUHSC-S IRB approval protocol ID H97-609 and The Affiliated First Hospital, Harbin Medical University Project ID: HYDYY-CK-KXYJ-0701. Informed consent for placental collection was waived by the IRB or Ethics Committees at both institutions because specimens and data were collected and analyzed retrospectively for research purposes only. Normotensive pregnancy was defined as normotension (the blood pressure < 140/90 mmHg), absence of proteinuria, obstetrical complication, and medical diseases. Pre-eclampsia was defined as a sustained blood pressure ≥ 140/90 mmHg on two separate measurements (previously normotension) with a urine dipstick protein of 1+ or greater, or a 24 h urine protein collection of ≥300 mg in the specimen. To avoid interference from clinical phenotypic differences in pre-eclamptic patients, patients whose situations were complicated by HELLP syndrome (hemolysis, elevated liver enzyme, and low platelet count), diabetes, and/or renal disease were excluded. No patients had autoimmune diseases. Smokers and patients with infections were also excluded from the study. The clinical characteristics of study subjects in the normal pregnant and pre-eclamptic groups are shown in Table 1.

### 2.3. Human Placenta Trophoblast Cells Isolation and Culture

Trophoblast cells from normal and pre-eclamptic placentas were isolated as previously described [14]. Briefly, trophoblast cells were isolated by digestion of placental villous tissue by 0.125% trypsin and 0.01% deoxyribonuclease, and further purified by Percoll gradient centrifugation. Isolated trophoblasts were incubated with DMEM supplemented with 10% FBS and antibiotics under standard culturing conditions at 37 °C with a 5% CO_2_ humidified atmosphere. For the measurement of cellular H_2_S levels and soluble H_2_S production, cells were seeded into 12-well plates with 2 × 10^6^ cells/well. Culture medium and cell lysis were collected at 24, 48, 72, and 96 h of culture, in triplicate for each time point. For the protein expression study, cells were seeded into 6-well plates with 5 × 10^6^ cells/well. Total cell protein was extracted after 72 h of culture.

### 2.4. Free H_2_S Measurement

For sample preparation, de-oxygenated Tris-HCl buffer (100 mM, pH 9.5) containing 0.1 mM diethylenetriamine pentaacetate (DTPA) was used to stabilize H_2_S in the medium and the cell lysate at the time of sample collection. Free H_2_S in the culture medium and cell lysate were measured by reversed-phase high performance liquid chromatography (RP-HPLC) as previously described [15]. Briefly, 50 μL monobromobimane (MBB) at a concentration of 10 mM, a fluorescent reagent, was added to each sample before the assay. MBB reacts with free H_2_S, forming stable products of thiosulfate bimane (TSB) and sulfide-dibimane (SDB), which were then measured by a RF-HPLC (Shimadzu Prominence 20A). The RF-HPLC was equipped with an Eclipse XDB-C18 column (4.6 × 250 mm, 80 Å) with gradient elution by 0.1% (*v*/*v*) trifluoroacetic acid in acetonitrile, and a fluorescence detector (model RF-10AXL) with excitation and emission wavelengths at 390 nm and 475 nm. The typical retention time for MBB and SDB was 15.75 min and 16.7 min, respectively. Free H_2_S concentration was calculated based on the standard curve generated with SDB at a concentration of 0.2 to 200 µM, and data are expressed as µM/2 × 10^6^ cells.

### 2.5. Immunohistochemistry

Villous tissue of freshly obtained placentas was fixed immediately with 10% formalin and embedded with paraffin. Tissue sections with thicknesses of 4 μm were used for immunostaining. A standard immunostaining procedure was performed as described previously [14]. Briefly, deparaffinization was performed with xylene and ethanol alcohol. Antigen retrieval was performed by boiling tissue slides with 0.01 mol/L citric buffer. Hydrogen peroxide (2%) in PBS was used to quench tissue endogenous peroxidase activity. After blocking with 5% goat serum (cat# 005-000-121, Jackson ImmunoResearch Inc., West Grove, PA, USA), tissue sections were incubated with primary monoclonal antibodies specific against human CBS (1:1000 dilution), CSE (1:200 dilution), and 3-MST (1:500 dilution) overnight at 4 °C. Subsequently, corresponding biotinylate-conjugated secondary antibodies (1:200, diluted in 1.5% blocking serum) and an ABC staining system (Vector Laboratories, Inc., Burlingame, CA, USA) were used following the manufacturer’s instruction. Stained slides were counterstained with Gill’s formulation hematoxylin. The sections not stained with a primary antibody were used as negative controls. Stained slides were reviewed under an Olympus microscope (Olympus IX71; Olympus, Japan), and images were captured with a digital camera linked to PictureFrame computer software (Uptronics Inc., Sunnyvale, CA, USA). In general, 3–4 images, under a 10× ocular lens and an objective lens with 40× magnification, were randomly captured by digital camera and recorded into a microscope-linked PC computer.

### 2.6. Protein Expression by Western Blot

Trophoblast expression of CBS and CSE were examined by Western blot as previously described [16]. Total cellular protein was collected after 72 h of culture. Equal total cellular protein, 10 µg/per sample, was used for electrophoresis, and then transferred onto nitrocellulose membranes. After blocking, the membranes were incubated with the primary monoclonal antibodies specific against human CBS (1:5000) and CSE (1:4000) overnight at 4 °C, followed by the matched secondary antibody (Bio-Rad, Hercules, CA, USA). The bound antibody was visualized with an enhanced chemiluminescent (ECL) detection kit (Amersham Corp, Arlington Heights, IL, USA). The band density of CBS, CSE, and β-actin for each sample was scanned and analyzed by NIH Image J2 software (Version 2.14.0/1.54f). β-actin expression was used as a loading control and normalized for each sample.

### 2.7. Statistical Analysis

Patient demographic and clinical data are expressed as mean ± SD. H_2_S production and protein expression data are presented as mean ± SE. Statistical analysis was performed with unpaired *t*-test, paired *t*-test, Chi-square test or AVOVA using Prism computer software version 10 (GraphPad Software, Inc., La Jolla, CA, USA). A probability of less than 0.05 was considered statistically significant.

## 3. Results

### 3.1. Demographic and Clinical Data of Study Subjects

Table 1 shows the demographic and clinical data for study subjects in normal and pre-eclamptic pregnancies. There was no statistical difference in maternal age, racial status, body mass index (BMI), gravidity and parity, or delivery mode between control and pre-eclampsia groups. While maternal systolic and diastolic blood pressure were significantly higher in the pre-eclamptic than in the control group. Gestational age at delivery, placental weight, and newborn weight were significantly lower in the pre-eclamptic group than in the control group. In the pre-eclampsia group, 11 out of 12 cases had newborn birth weights less than the 50th percentile (<50% = 3, <25% = 3, <10% = 5). There was no difference in newborn gender between the two groups, as seen in Table 1.

### 3.2. H_2_S Production and Cellular Levels in Trophoblasts from Normal and Pre-Eclamptic Pregnancies

**Figure 1:**
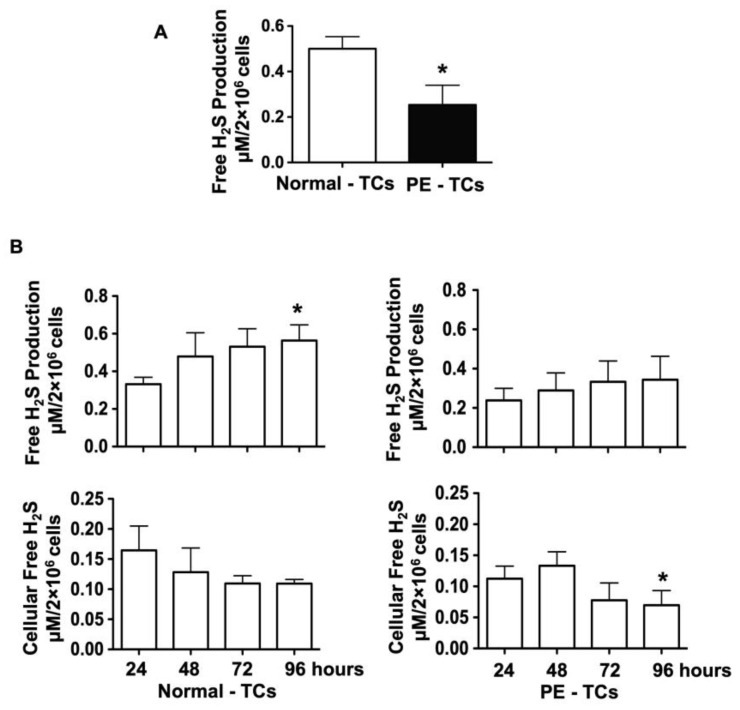
Free H_2_S production in the culture medium and cell lysate in trophoblast cultures from normal and pre-eclamptic groups. (**A**) Free H_2_S production measured in the cell culture medium. Free H_2_S production was significantly reduced in placental trophoblasts from pre-eclamptic (*n* = 5), compared to normal (*n* = 8), pregnancies, * *p* < 0.05. (**B**) Free H_2_S production and free cellular H_2_S levels in placental trophoblasts cultured at 24, 48, 72, and 96 h in normal (*n* = 4) and pre-eclamptic (*n* = 4) cases. Free H_2_S production was increased in trophoblasts from normal cases, * *p* < 0.05 (96 h. vs. 24 h). On the other hand, intracellular free H_2_S levels were reduced in trophoblasts from 24 to 96 h of culture. The increasing of free H_2_S production and reduction of intracellular free H_2_S levels occurred in a time-dependent manner. Although similar patterns of increased free H_2_S production and reduced intracellular free H_2_S levels were also observed in placental trophoblasts from pre-eclamptic cases, the degree of free H_2_S production and intracellular free H_2_S levels were much lower in trophoblasts from pre-eclamptic cases than from normal cases. * *p* < 0.05 (96 h vs. 24 h).

We first tested if placental trophoblasts produce H_2_S. Trophoblasts were isolated from freshly delivered placentas from eight normal and five pre-eclamptic pregnancies and cultured for 4 days. Medium specimens were collected at the end of experiment and H_2_S concentrations were measured. Our results showed that the medium H_2_S concentration was significantly lower in trophoblast cultures in the pre-eclamptic group than in the control group, with 0.301 ± 0.093 µM/2 × 10^6^ cells vs. 0.500 ± 0.053 µM/2 × 10^6^ cells, *p* < 0.05 (Figure 1A). Among the 13 cultures, medium specimens and cell lysate were also collected at 24, 48, 72, and 96 h of incubation from four normal and four pre-eclamptic cases and free H_2_S levels in the medium and cell lysate were measured. As shown in Figure 1B, medium H_2_S levels were increased in trophoblast cultures in the control group, * *p* < 0.05. In contrast, intracellular free H_2_S levels were reduced from 24 to 96 h of culture. Free H_2_S production increased and intracellular H_2_S levels were reduced in a time-dependent manner. Similar patterns of increased H_2_S production and reduced intracellular free H_2_S levels were also observed in trophoblast cultures in the pre-eclamptic group. However, the magnitude of H_2_S production and intracellular H_2_S levels were much less in trophoblast cultures in the pre-eclamptic group than in the normal group.

### 3.3. Expression of CBS, CSE, and 3-MST in Placental Villous Tissue from Normal and Pre-Eclamptic Pregnancies

**Figure 2:**
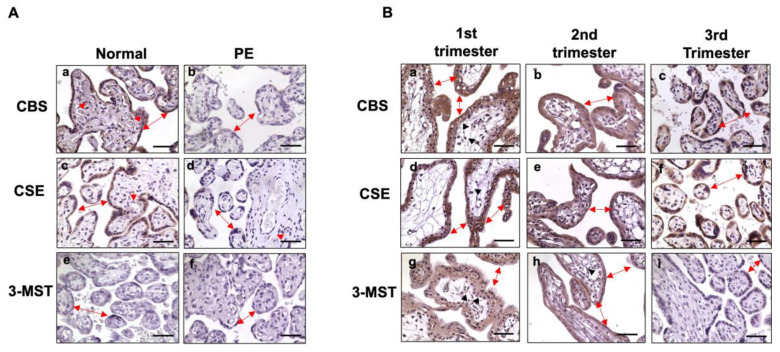
Expression of CBS, CSE, and 3-MST, as determined by immunostaining in placental villous tissue. (**A**) Representative CBS, CSE, and 3-MST expression, detected by immunostaining from normal and pre-eclamptic cases. In the normal group, both CBS and CSE were detected in the syncytiotrophoblast layer (double arrow). CBS, but not CSE, was also detected in the villous core fetal vessel endothelium (solid arrowhead). Expression of CBS and CSE were markedly reduced in syncytiotrophoblasts in pre-eclamptic cases (Figure 2**A**(b,d)) compared to controls. Expression of 3-MST was undetectable in placental specimens from both normal and pre-eclamptic cases (Figure 2**A**(e,f)). Bar = 50 µm. (**B**) Representative CBS, CSE, and 3-MST expression in the first, second, and third trimester placental specimens. Both CBS and CSE are strongly expressed in cyto- and syncytio-trophoblasts in the first, second, and third trimester placentas. CBS: **a**, **b**, and **c**; CSE: **d**, **e**, and **f**, respectively. Expression of 3-MST was detected in the first (**g**) and the second (**h**) trimester placentas, but not in the third/term (**i**) placentas. Bar = 50 µm.

Next, we examined the three key H_2_S converting enzymes CBS, CSE, and 3-MST in placental tissues from normal (*n* = 7) and pre-eclamptic (*n* = 7) pregnancies. Figure 2A shows representative CBS, CSE, and 3-MST expression, as determined by immunostaining. In the normal group, CBS and CSE were mainly present in syncytiotrophoblasts, as can be seen in Figure 2A(a,c). Expression of CBS, but not CSE (Figure 2A(c)), was also detected in villous core fetal vessel endothelium (Figure 2A(a)). In comparison, trophoblast expression of CBS and CSE (Figure 2A(b,d)) was barely detected or much reduced in trophoblasts in the pre-eclamptic group. CBS expression was not seen in villous core fetal vessel endothelium in placentas from pre-eclamptic cases (Figure 2A(b)). Interestingly, 3-MST expression was undetectable in placental villous tissue from both normal and pre-eclamptic cases, as can be seen in Figure 2A(e and f), respectively.

As noted in Table 1, patients in the pre-eclamptic group were delivered early compared to those in the normal group. To determine if reduced CBS and CSE expression in placental trophoblasts in the pre-eclamptic group is related to early gestation age at delivery and if placental trophoblasts express 3-MST, we then examined CBS, CSE, and 3-MST expression in villous tissue from first and second trimester placentas. As shown in Figure 2B, robust CBS and CSE signals were detected in first trimester (6–8 weeks, *n* = 3), second trimester (16–20 weeks, *n* = 3), and third trimester placentas from normal pregnancies. Interestingly, strong 3-MST signals were also detected in the first and second trimester placentas, but not in the third/term trimester placentas. Expression of CBS, CSE, and 3-MST was not only seen in cyto- and syncytiotrophoblasts, but also noticed in villous stromal cells in first and second trimester placentas (Figure 2B). These results revealed that CBS and CSE are strongly expressed in placental trophoblasts throughout pregnancy in normotensive pregnancies, suggesting that CBS and CSE expression in placental trophoblasts is not dependent on gestational age. The evidence of 3-MST presented in first and second trimester placentas, but not in third trimester/term placentas, indicates that 3-MST could be dependent on gestational age or development, and may not be responsible for the reduced H_2_S production in placental trophoblasts from pre-eclamptic pregnancies. Representative images of negative control immunostaining for CBS, CSB, and 3-MST expression in the first, second, and third trimesters or term placentas are presented in Supplementary Figure S1.

### 3.4. Downregulation of CBS and CSE Expression in Placental Trophoblasts from Pre-Eclamptic Pregnancies

**Figure 3:**
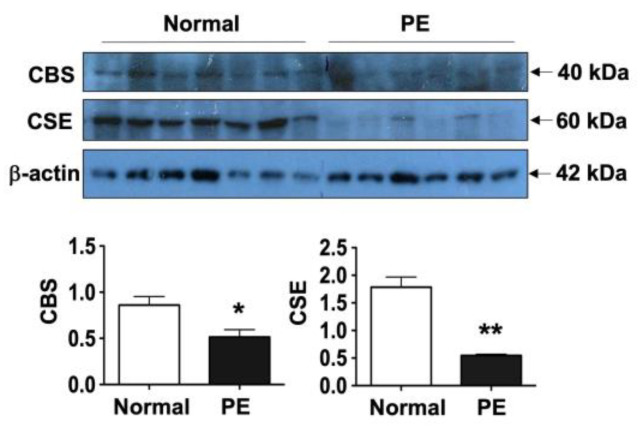
Trophoblast CBS and CSE protein expression by Western blot. Consistent with immunostaining results, the levels of CBS and CSE protein expression were also significantly reduced in trophoblasts from pre-eclamptic (PE, *n* = 6) compared to those from normal (*n* = 7) cases. The bar graphs show relative protein expression of CBS and CSE after normalization with β-actin expression for each sample, * *p* < 0.05 and ** *p* < 0.01: PE vs. normal, respectively.

To further confirm reduced CBS and CSE expression in placental trophoblasts from pre-eclamptic vs. normal pregnancies, we also examined protein expression of CBS and CSE by Western blot analysis. As shown in Figure 3, consistent with immunostaining results, the amount of CBS and CSE protein expression were also significantly reduced in placental trophoblasts from pre-eclamptic compared to normal pregnancies. The bar graphs show the mean for relative protein expression of CBS and CSE after normalization with β-actin expression in each sample. Expression of CBS and CSE in trophoblasts from pre-eclamptic cases was significantly reduced compared to those from normal cases; * *p* < 0.05 and ** *p* < 0.01, respectively. Since 3-MST was undetectable in tissue sections from normal and pre-eclamptic placentas, trophoblast 3-MST expression was not determined by Western blot.

## 4. Discussion

To the best of our knowledge, this is the first study to evaluate free H_2_S production, cellular H_2_S levels, and the three major H_2_S converting enzymes, CBS, CSE, and 3-MST, in primary placental trophoblasts from normal and pre-eclamptic pregnancies. We found that placental trophoblasts produce H_2_S, and cellular H_2_S levels, along with H_2_S production, were significantly reduced in placental trophoblasts from pre-eclamptic compared to cells from normal cases. We further found that expression of CBS and CSE was significantly reduced in placental trophoblasts from pre-eclamptic cases compared to normal cases. However, 3-MST was neither detectable in normal placental villous tissues, nor in pre-eclamptic cases.

It is well-known that H_2_S exerts vasodilative, antioxidative, and anti-inflammatory activities. H_2_S has been well-studied in the cardiovascular system. H_2_S could promote angiogenesis, trigger vasorelaxation, reduce blood pressure and inflammation, and limit atherosclerosis, as well as confer cardio protection [17,18,19]. Reduced H_2_S production or altered CBS and CSE expression were reported in cardiovascular related diseases and disorders [20,21]. Pre-eclampsia is a hypertensive disorder in human pregnancy. Although, in the present study, we did not explore how CBS and CSE expression is regulated in placental trophoblasts and the reason for CBS and CSE downregulation in trophoblasts from pre-eclamptic pregnancies, there is no doubt that downregulation of CBS and CSE expression could lead to reduced H_2_S production in pre-eclamptic placentas, and consequently, reduced H_2_S production may contribute to trophoblast dysfunction, including increased vasoconstriction, increased oxidative stress, and increased inflammatory responses in pre-eclampsia. Results from an animal study support the notion that aberrant H_2_S production may contribute to the pathogenesis of pre-eclampsia; in that study, the investigators found that H_2_S could ameliorate pre-eclampsia via suppression of toll-like receptor 4-activated inflammation in the rostral ventrolateral medulla [22].

As noted, the mean gestational age at delivery in the pre-eclampsia group was less than 34 weeks. To determine if reduced CBS and CSE expression or H_2_S production by placental trophoblasts in pre-eclampsia are related to the lower gestational age at delivery, and whether trophoblasts express 3-MST, we further examined CBS, CSE, and 3-MST expression in villous tissues from first and second trimester placentas. As shown in Figure 2B, both CBS and CSE were strongly expressed in the first, second, and third trimester placentas. Strikingly, strong 3-MST signals were also detected in the first and second trimester, but not in the third trimester/term, placentas. These data provide reasonable evidence that CBS and CSE are present, in humans, in placental trophoblasts throughout pregnancy, and the downregulation of CBS and CSE expression seen in trophoblasts from pre-eclamptic cases may not be due to preterm birth. However, the presence of 3-MST in first and second trimester placentas, undetectable in the third trimester placentas suggest that 3-MST expression in placental trophoblasts is likely developmentally dependent. Our observation of both CBS and CSE in the first and second trimester placental trophoblasts is different from what was reported previously; in that study [11], BeWo and HTR-8/SVneo cells were used to study the effects of trophoblast-derived H_2_S on placental artery endothelial angiogenesis, and the results showed that HTR-8/SVneo cells expressed CSE, but not CBS [11]. The difference in H_2_S-converting enzyme expression between first trimester placental trophoblasts and HTR-8/SVneo cells suggests that functional variation may exist between primary placental trophoblasts and immortalized or transformed trophoblast cells. In fact, the differences in primary isolated trophoblasts from HTR-8/SVneo cells or other transformed trophoblast-derived choriocarcinoma cells have been reported, such as wide-ranging differences in DNA methylation [23] and miRNA expression profiles [24].

CBS and CSE are widely expressed as key enzymes in the reverse trans-sulfuration (RTS) pathway, by which methionine (Met) is recycled to cysteine, while 3-MST generates sulfide from 3-mercaptopyruvate (3-MP), a product of cysteine deamination. 3-MST is localized in mitochondria as well as the cytoplasm and mediates the reaction of 3-mercaptopyruvate with dihydrolipoic acid and thioredoxin to produce H_2_S. Expression of CBS and CSE was reported be to tissue specific, with CBS being expressed predominantly in the brain, and CSE in peripheral tissues, including lung tissue, and the expression and activity of CSE were found to be developmentally regulated [25]. 3-MST expression also varies in a cell-specific manner. A study conducted by Tomita et al. showed that 3-MST is present in the brain, liver, kidneys, testis, and endocrine organs, but not in the lungs, spleen, thymus, and small intestines in rats [26]. Similar organ or tissue distributions of 3-MST in mouse were also reported [27]. An animal study conducted by de la Cruz et al. also found that exogenous H_2_S could restore CBS and CSE, but not 3-MST protein expression in the hypothalamus and brainstem after severe traumatic brain injury [28]. The difference in 3-MST expression in placental trophoblasts between first/second and third trimester placentas that we found in the present study also provides reasonable evidence that the role of 3-MST in placental trophoblasts might be different from CBS and CSE.

## 5. Conclusions

In summary, our study demonstrated that placental trophoblasts produce H_2_S and both free H_2_S production and cellular H_2_S levels are significantly reduced in trophoblasts from pre-eclamptic placentas, compared to the cells from normal placentas. Our data further revealed that reduced H_2_S production was very likely due to downregulation of CBS and CSE expression in trophoblasts from pre-eclamptic placentas. The novel finding of our study is the gestational age dependent expression of 3-MST in placental trophoblasts; i.e., strong 3-MST expression is seen in the first and second, but not in the third/term, placentas, which is different from CBS and CSE, as both are robustly expressed in placental trophoblasts throughout normal pregnancies, suggesting the regulation and function between 3-MST and CBS/CSE are different in placental development. There are, however, several limitations to this study. As mentioned earlier, we did not explore how CBS and CSE expression are regulated in placental trophoblasts and what the mechanism(s) of downregulation of CBS and CSE expression in placental trophoblasts in pre-eclampsia is/are. It is also not known if labor has any effect on trophoblast H_2_S production, nor whether aberrant trophoblast H_2_S production has any effects on placental growth or fetal development. Currently, little is known about the function of 3-MST in trophoblasts. How the 3-MST pathway is regulated during placental development warrants further investigation.

## Figures and Tables

**Table 1:** Demographic data for placentas used in the study.

Variables	Normal (*n* = 18)	Pre-Eclampsia (*n* = 12)	*p* Value
Maternal Age	29 ± 6	29 ± 7	0.938
Racial Status ¶ White Black Other			
	5 12	2 9	0.6693
	1	1	
BMI	36.4 ± 6.2	37.9 ± 9.5	0.593
Blood Pressure (mmHg) Systolic Diastolic	120 ± 9 67 ± 9	171 ± 12 99 ± 15	<0.001 <0.001
Primigravida ¶	2	3	0.3644
Gestational Age (weeks^+days^) at Delivery	38^+3^ ± 1^+2^	33^+0^ ± 3^+4^	<0.001
Delivery Mode ¶ Vaginal Delivery C-Section			
	8	3	0.2789
	10	9	
Fetal Gender ¶ Male Female	7 11	6 6	0.7106
Baby Weight (gram)	3341 ± 565	1832 ± 778	<0.001
Placental Weight (gram)	619 ± 131	402 ± 137	0.001

BMI: body mass index; data are expressed as mean ± SD. ¶ data were analyzed by Chi-square test.

## Data Availability

Data are available upon request.

## Supplementary Materials

The following supporting information can be downloaded at https://www.mdpi.com/article/10.3390/pathophysiology32010010/s1. Figure S1: Representative images of negative control for CBS, CSE, and 3-MST immunostaining in villous tissue from the first, second, and third trimester placentas. Bar = 50 µm.
