# Supplementary material for: Altered Production and Cellular Levels of Hydrogen Sulfide (H2S) in Placental Trophoblasts from Pregnancies Affected by Pre-Eclampsia
Source: Pathophysiology. 2025 Mar 4;32(1):10. doi: 10.3390/pathophysiology32010010 (PMC11946082; doi:10.3390/pathophysiology32010010)
Supplement: Supplementary file 1 [file pathophysiology-32-00010-s001.zip › pathophysiology-3357063-supplementary.pdf]

Altered Production and Cellular Levels of Hydrogen Sulfide (H_2S) in Placental Trophoblasts from Pregnancies Affected by Pre-eclampsia

Xiaodan Chu [1,2], Jie Xu [1], Xinggui Shen [3], Wenji Sheng [1,4], Jingxia Sun [4], Yang Gu [1], David F. Lewis [1], Danielle Cooper [1], Dani Zoorob [1] and Yuping Wang [1,*]

[1] Department of Obstetrics and Gynecology, Louisiana State University Health Sciences Center,
Shreveport, LA 71103 USA; 820715@hrbmu.edu.cn (X.C.); jxu001@lsuhs.edu (J.X.); 813309@hrbmu.edu.cn (W.S.); yg626@yahoo.com (Y.G.); dfl001@lsuhs.edu (D.F.L.); dsc001@lsuhs.edu (D.C.); dzo001@lsuhs.edu (D.Z.); ywa001@lsuhs.edu (Y.W.)

[2] Department of Obstetrics and Gynecology, The Second Affiliated Hospital, Harbin Medical University, Harbin, 150086, China

[3] Department of Pathology, Louisiana State University Health Sciences Center, Shreveport, LA 71103, USA; xsh001@lsuhs.edu

[4] Department of Obstetrics and Gynecology, The First Affiliated Hospital, Harbin Medical University,
Harbin 150001, China; sjxsw2020@szu.edu.cn

* Correspondence: ywa001@lsuhs.edu; Tel.: +318-675-5370

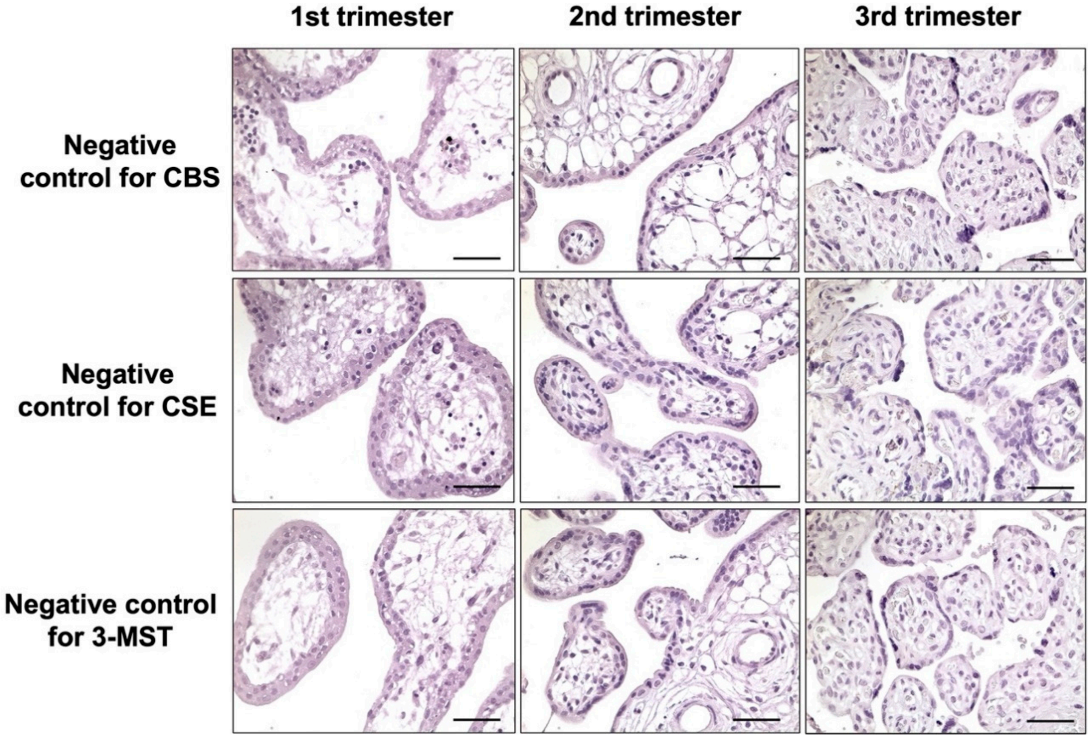

Supplementary Figure S1. Representative images of negative control for CBS, CSE, and 3-MST immunostaining in villous tissue from the first, second, and third trimester placentas. Bar = 50 μm.
